# Editorial: State-of-the-art muscle physiology research: from single molecules and cells to living organisms

**DOI:** 10.3389/fphys.2025.1636988

**Published:** 2025-06-17

**Authors:** Norio Fukuda, Madoka Suzuki, Kotaro Oyama, Norika Liu

**Affiliations:** ^1^ Department of Molecular Physiology, The Jikei University School of Medicine, Tokyo, Japan; ^2^ Department of Cell Physiology, The Jikei University School of Medicine, Tokyo, Japan; ^3^ Institute for Protein Research, The University of Osaka, Osaka, Japan; ^4^ Takasaki Institute for Advanced Quantum Science, National Institutes for Quantum Science and Technology, Gunma, Japan; ^5^ The International Research Center for Medical Sciences, Kumamoto University, Kumamoto, Japan

**Keywords:** embryology, heart, mavacamten, myocardium, myosin, nano-imaging, sarcomere, titin

Since the turn of the 21st century, scientists have applied various optical and molecular biological technologies to ascertain critical details on the structure and function of skeletal and cardiac muscles in health and disease. As such, the newly developed technologies will further deepen our understanding of the molecular and cellular mechanisms of striated muscle contraction and relaxation, thereby providing new prospective means to facilitate the diagnosis and treatment of various diseases, especially in the heart. This Research Topic covers recent advances in the studies on the hierarchical structure and function of skeletal and cardiac muscles, as well as the pre- and post-natal development of the heart.

James A. Spudich received the Albert Lasker Basic Medical Research Award in 2012 for his trailblazing investigations of the molecular motors that drive our skeletal muscle contractions and heartbeats. Spudich is highly regarded for his work on the elucidation of precise mechanisms of energy transduction by myosin molecules when interacting with actin filaments. Notably, his group developed two important single molecule experimental systems, both of which are now commonly used worldwide under various conditions by utilizing various types of proteins; first, an *in vitro* motility assay enabling the measurement of the velocity of individual F-actin sliding on myosin molecules, and second, a laser trap assay enabling the measurement of the step size as well as the amount of force produced by one myosin molecule interacting with a single F-actin. After successfully elucidating the mechanism of actomyosin energy transduction, the Spudich group became interested in the mutants of human β-cardiac myosin involving cardiac disease. As described in Spudich, hypertrophic cardiomyopathy (HCM) is a genetic disease that affects 1 in 500 to 1 in 200 individuals. HCM is characterized by left ventricular hypertrophy, resulting in a decreased volume of the left ventricle and accordingly a reduction of stroke volume. HCM is associated with mutations in genes encoding various thick and thin filament proteins, but most mutations occur in either *MYH7* or *MYBPC3*, encoding human β-cardiac myosin heavy chain and cardiac myosin binding protein-C. Considering these experimental findings, Spudich became a member of the discovery board of SmithKline Beecham Ltd., and posed the following question: “Why not target the heart’s sarcomeric cytoskeletal proteins directly?” With this question in mind, he was involved in the foundation of Cytokinetics, Inc., the first biotech company to target cytoskeletal molecular motors, and later, the foundation of MyoKardia, Inc. At MyoKardia, Inc., Spudich and people with various backgrounds found the compound, mavacamten, that reduces the power output of the heart by binding to cardiac myosin. They tested mavacamten on HCM mouse models, and found that the compound decreases cardiac contractility, suppresses the onset of hypertrophy and cardiomyocyte disarray, and, surprisingly, reverses these HCM-induced changes when the mice were treated after the changes had been induced. Then, after highly successful phase 3 clinical trials, MyoKardia, Inc. was acquired by Bristol Myers Squibb Company in 2020, and in 2022 they received approval from the U.S. Food and Drug Administration for mavacamten. In 2023, mavacamten received the honorable Prix Galien USA award, the country’s preeminent prize acknowledging the leading-edge of scientific advances in life sciences. The key to mavacamten’s success was having a deep understanding of the actin-activated myosin system and the insight to target the downstream effector, minimizing pleiotropic effects that often lead to toxicity. In light of global uncertainties for the environment of basic scientists, especially young and mid-level scientists, this article by a Lasker Award winner provides strong proof for why basic science research deserves to be funded, especially because public funding is provided in many countries with the assumption that basic science research will eventually lead to improvements in healthcare.

Titin (connectin), the largest protein expressed in mammals known to date (molecular weight, ∼3–4 MDa), is the third filament in the striated muscle sarcomere, extending from the Z-line to the M-line where the N- and C-termini are anchored, respectively. Titin functions as a molecular spring by generating passive force in cardiac sarcomeres via predominantly three extensible segments in the I-band; i.e., the tandem immunoglobulin (Ig)-like domain, PEVK domain and N2B domain (e.g., Granzier and Labeit, 2004; Fukuda et al., 2008; Fukuda et al., 2010 and references therein). In addition, the binding of chaperones to the N2A domain also regulates its spring-like properties. In the heart, elastic segments function as a spring that supports early diastolic recoil and late diastolic resistance to stretch. Stroik et al. discuss in detail the structure and functions of titin’s various domains (as described above), as well as how titin splicing (i.e., N2B titin vs. N2BA titin) and post-translational modifications contribute to these functions under various conditions. The first evidence for titin phosphorylation was provided in the early 2000s by the Granzier group who demonstrated that the N2B segment of both N2B titin and N2BA titin can be phosphorylated by protein kinase A (PKA), causing a substantial decrease in passive force in intact and skinned cardiac preparations (Yamasaki et al., 2002; Fukuda et al., 2005). This is a good example of an adaptive response of cardiac sarcomeres to changes in the cardiovascular system to enhance ventricular filling during reduced diastolic time under β-adrenergic stimulation. Efforts are now being made to fully characterize the phosphorylation/dephosphorylation of titin by various kinases, as well as signaling pathways at titin’s various segments, towards understanding of the overall picture of post-translational modifications of titin. Finally, Stroik et al. provide useful information on titin and heart diseases, such as dilated cardiomyopathy (DCM), heart failure with preserved ejection fraction (HFpEF) and diabetic cardiomyopathy (DbCM). Of note, titin truncating variants (TTNtvs) represent the most common cause of familial and sporadic DCM, accounting for upwards of 25% of familial cases and ∼15% of sporadic cases. Surprisingly, TTNtvs are also found in ∼1–2% of the general population. Pathogenic TTNtvs are typically carried in the heterozygous state and are over-represented in the A-band. Considering the fact that titin has many physiologically important functions, the molecule can potentially be an appealing therapeutic target for various types of heart diseases.

It has long been perceived that sarcomeres in cardiac and skeletal muscles shorten and lengthen in synchrony during excitation-contraction coupling (as discussed in detail in Kurihara and Fukuda, 2024). Kobirmaki-Shimozawa et al. logically pose an amendment to this traditional view based on their previous experimental findings, in that the lengths of individual sarcomeres vary at rest and do not change in a synchronous fashion throughout the contraction cycle, even along the same myofibrils. In earlier studies, these authors established high-speed, high-resolution nano-imaging technologies for cardiac sarcomeres in living mice (e.g., Kobirumaki-Shimozawa et al., 2016; Kobirumaki-Shimozawa et al., 2020; Shimozawa et al., 2017). More recently, they successfully developed a quantification method for the magnitude of synchrony of sarcomere dynamics by introducing the novel parameter “Contribution Index (CI),” defined based on correlation coefficient matrices between individual sarcomere movements along myofibrils in ventricular myocytes in living mice [from −1 (complete negative correlation) to 1 (complete positive correlation)] (Kobirumaki-Shimozawa et al., 2021). It is important that CI between an individual sarcomere and the average of all sarcomeres along a myofibril is within the range between ∼0.3 and ∼0.5 under healthy conditions, of which values clearly decrease in association with a reduction in ventricular pressure. It is therefore indicated that sarcomere synchronization plays an important role in the regulation of myocardial contractility. Because sarcomeres are connected in series along a myofibril, and their lengths usually vary even during diastole (as in Kobirumaki-Shimozawa et al.), the movements of adjacent sarcomeres with different lengths *inevitably* become asynchronous. This is because cardiac titin isoforms (especially N2B titin) are shorter compared with their skeletal counterparts; therefore, upon systole, sarcomeres longer than their adjacent neighbors by merely, e.g., ∼0.1 μm shorten to a greater extent and stretch these neighbors by taking advantage of the Frank-Starling mechanism that has been proven to strictly operate at the single sarcomere level (Kobirumaki-Shimozawa et al., 2021). It is well known that impaired systolic function results in an increase in the ventricular volume due to blood congestion, such as in dilated cardiomyopathy or congestive heart failure. Because a high level of titin-based passive force will be generated under these stretched conditions, sarcomeres will be under *dynamic instability* along myofibrils due to enhanced tug-of-war between adjacent neighbors, which will ultimately cause marked asynchrony that can progressively exacerbate myocardial contractility. To fully understand the mechanisms of cardiac disorders, therefore, it is important to analyze not only protein expression profiles using traditional methodologies but also sarcomere dynamics as well as inter-sarcomere interactions at nm precision under living conditions in future studies.

In the field of muscle physiology and biophysics, myofibrillogenesis has gained a lot of attention from many scientists by taking advantage of various technologies. It has indeed been established that titin plays an important role in this process as a molecular template, especially for thick filament remodeling and distribution (see, e.g., Udaka et al., 2008 and references therein). From the macroscopic viewpoint, the precise mechanisms of the morphogenesis of the heart are yet to be fully elucidated; our knowledge is limited on how the heart effectively exerts pump functions during the course of maturation, especially in mammals. Liu and Nakano review this important process, and propose a novel mechanism focusing on endocardial hematopoiesis during the maturation process of the heart. It is known that dramatic transformations occur during remodeling in the internal structure of the heart; namely, following cardiac looping, endocardial cells in the outflow tract and atrioventricular canal regions undergo endothelial-to-mesenchymal transformation (EndoMT) to form cushion mesenchyme that eventually remodel into cardiac valves and septum. Their recent studies demonstrated that the endothelial-to-hematopoietic transformation (EHT) occurs during remodeling (Nakano et al., 2013; Liu et al., 2023). Based on experimental findings, they have proposed the following two mechanisms. First, Nkx2-5/Notch signaling drives the transformation of endocardial cells into both mesenchymal cells (via EndoMT) and hematopoietic cells (via EHT). Second, hematopoietic cells derived from the endocardium express *Dhrs3* and suppress the retinoic acid signaling to promote differentiation of these cells into macrophages that contribute to the formation of mature heart valves. However, still data images (typically, Figure 1 in Liu and Nakano) are insufficient to provide complete mechanistic implications at the molecular and cellular levels on how, for instance, macrophages operate in the formation of heart valves. Therefore, as these authors discuss, high-spatial and high-temporal imaging technologies (as in Kobirumaki-Shimozawa et al., 2016; Kobirumaki-Shimozawa et al., 2020; Kobirumaki-Shimozawa et al., 2021; Shimozawa et al., 2017) need to be employed in future studies to fully uncover how the hematopoietic endocardium contributes to heart development and systemic hematopoiesis.

In summary, this Research Topic exemplifies how state-of-the-art technologies have unveiled the elaborate systems in skeletal muscle and the heart. We must move forward to further elucidate the structural and functional hierarchy of striated muscles, not only by taking advantage of the established technologies presented in this Research Topic, but also by developing newer advanced technologies. In doing so, all the contributions made by the authors here will be integrated for the prevention, diagnosis and treatment of various skeletal muscle and heart diseases.
